# A Trait-State Model of Trust Propensity: Evidence From Two Career Transitions

**DOI:** 10.3389/fpsyg.2019.02490

**Published:** 2019-11-05

**Authors:** Lisa van der Werff, Yseult Freeney, Charles E. Lance, Finian Buckley

**Affiliations:** ^1^DCU Business School, Irish Institute of Digital Business, Dublin City University, Dublin, Ireland; ^2^DCU Business School, Dublin City University, Dublin, Ireland; ^3^Department of Psychology, University of the Western Cape, Cape Town, South Africa

**Keywords:** trust, trust propensity, socialization, career transition, trait-state-occasion models, cognitive depletion, latent growth model, personality change

## Abstract

Trust propensity is typically conceptualized as a stable, trait-like, exogenous variable. Drawing on the social investment principle of personality change, we argue that trust propensity has situationally specific components and is likely to be less stable during periods of career transition. Using a latent curve-latent state-trait model, we present evidence that suggests that trust propensity has stable (trait) and unstable (state) components during career transition periods and that it has the potential to change over time. Our results are replicated across two, transitional workplace populations during a process of (re)socialization into an organization. In our second study, we also expand our focus to examine correlates of trust propensity and demonstrate the relationship between state and trait trust propensity and cognitive depletion. Our paper significantly extends knowledge of the nature of trust propensity and raises questions about the stability of this construct, one of the core tenets of trust theory.

## Introduction

The importance of trust as an enabler of positive workplace interactions has been established through a considerable body of empirical evidence and meta-analyses (Dirks and Ferrin, 2002; Colquitt et al., 2007; De Jong et al., 2017). Trust is commonly defined as a willingness to be vulnerable based on positive expectations of another party (Rousseau et al., 1998). The bases of these positive expectations are typically portrayed as a combination of trustor characteristics with perceptions of the trustworthiness characteristics of the other party (e.g., Mayer et al., 1995; McKnight et al., 1998). Since the seminal theory development of the mid-1990s, the empirical trust literature has focused predominantly on the examination of trustworthiness as the key driver of trust decisions, with very little attention given to the role of trustor characteristics. Trustor characteristics in the form of trust propensity (TP) plays a key role in the majority of trust theory however, the literature discussing its conceptualization and influence remains significantly underdeveloped (Schoorman et al., 2007; Frazier et al., 2013).

While TP has rarely been a central focus in empirical trust work, meta-analysis suggests that TP is a consistent predictor of trust across a range of interpersonal relationships (Colquitt et al., 2007). The small body of research that takes a closer look suggests TP is particularly influential in situations with high levels of uncertainty or ambiguity, such as new relationships (Gill et al., 2005; Alarcon et al., 2016; van der Werff and Buckley, 2017). Studies of TP have implicitly or explicitly assumed that it is a stable dispositional trait which does not change over time, with consistent definitions of TP as a stable factor across a range of studies (e.g., Mooradian et al., 2006; Ashleigh et al., 2012; Alarcon et al., 2016). The assumption of stability has influenced the, admittedly scarce, empirical study of TP in organizations in two key ways. First, as TP is not expected to change over time, it has been measured cross-sectionally, even in longitudinal studies of trust (e.g., Ferguson and Peterson, 2015; Alarcon et al., 2016; van der Werff and Buckley, 2017). Second, TP has been examined only as an antecedent in relationships, with no consideration that relational outcomes and the social contexts in which they occur may influence ongoing development of TP. One notable exception to this is a recent exploration of daily fluctuations in TP as influenced by the behavior of colleagues and fairness in the work environment (Baer et al., 2018a). Evidence for these daily fluctuations suggests the cross-sectional study of TP as a solely exogenous variable has been unwarranted but the literature has not yet explored the potential for meaningful and lasting change in TP over time.

Recent trends in the personality literature recognize the potential for change in personality traits across the lifespan (Roberts et al., 2006), during key transitionary periods (Boyce et al., 2015), as well as daily fluctuations in “personality states” over shorter time periods (Judge et al., 2014; Baer et al., 2018a). Longer term change during transitionary periods is particularly meaningful as societal level changes in expectations around the stability of work mean that the number of transitions an individual can expect to encounter during their working life has increased (Fouad and Bynner, 2008). Drawing on the social investment principle (Lodi-Smith and Roberts, 2007), we argue that situationally specific components of TP will vary during important career transitions. The aim of our study is to examine the trait and state aspects of TP during two key manifestations of social role investment, where individuals are taking on a new social role. Study 1 investigated the level of stability in TP as 195 young adults began their career as accountants and took on the social role of new employees. Study 2 builds on our first study by examining the level of stability in TP in a more occupationally heterogeneous sample at a different life stage and focuses on the transition of 247 working mothers back into the workplace following a substantial period of maternity leave. Within this second study, we also employ a different operationalization of TP to ensure the patterns of instability that we observe are not scale specific, and expand our focus to examine the impact of TP on cognitive depletion.

Our paper significantly extends knowledge of the nature of TP and raises questions about one of the core tenets of trust theory. This is particularly salient for any trust research that focuses on new relationships or ambiguous situations. For instance, empirical studies of trust in the context of career transitions, such as newcomer socialization, new team formation and organizational mergers, should consider the potential role of these transitions in influencing TP and the role of change in TP in influencing ongoing employee behavior. We also contribute to the personality literature in applying the social investment principle to the lower level facet trait of TP and adding to a very small body of literature that investigates changes in personality over a short timeframe. We further contribute to the personality literature in highlighting that changes in a trait can occur during work transitions but, importantly, that these shifts are not mere fluctuations and that new levels of a personality trait can endure beyond the initial change period. Finally, we make a methodological contribution to the applied psychology literature by demonstrating the utility of Tisak and Tisak’s (2000) latent curve-latent state-trait (LC-LST) model in one of its first applications in the organizational sciences.

## Theoretical Development

Theoretical exploration of trustor characteristics that influence trust have focused on the idea that some individuals may be more likely to trust than others. TP is a term used to describe this dispositional tendency or willingness to trust. The concept was first examined in detail by Rotter (1967, 1971), who introduced dispositional trust as an expectancy or attitude that others can be relied on which can be generalized from one party to another. In the wider social sciences, scholars have distinguished between thick, particularized trust, which is specific to a relationship, and thin, generalized trust which is more diffuse and directed at a wider circle of unfamiliar others (Delhey et al., 2011). Nannestad (2008, p. 428) describes generalized trust as “the bedrock of cooperation,” critical for social and economic transactions.

In models of trust commonly cited in the organizational psychology field, TP is typically positioned as a direct antecedent of trust as well as a moderator of the relationship between trustworthiness and trust (e.g., Mayer et al., 1995). Empirical research has generally supported this and established TP as an important contributor to workplace outcomes. Colquitt et al. (2007) argue that TP is the key driver of the cognitive leap necessary to engage in trust and a lens through which the trustworthiness of others can be judged (Govier, 1994). In their meta–analysis, Colquitt et al. (2007) demonstrate that TP impacts job performance concepts such as organizational citizenship behavior and counterproductive work behavior indirectly via trust as well as directly. Mayer et al. (1995) propose that this propensity is particularly influential in circumstances where information about the other party is not yet available, such as new relationships. Longitudinal studies of trust support this proposition and suggest that TP may be more important at the beginning of a relationship (van der Werff and Buckley, 2017) and with unfamiliar other parties (Alarcon et al., 2016). In line with this, research demonstrates that TP has a greater influence on trust when information about the other party is ambiguous (Gill et al., 2005) or when communication frequency is low (Becerra and Gupta, 2003). Given the dynamic qualities of many organizations in modern society, TP is growing in significance as employees are required to interact regularly with new, unfamiliar coworkers and clients, often in the absence of face to face contact (Delhey et al., 2011; Frazier et al., 2013).

Thus far, in the applied psychology literature, TP has been viewed as a trait-like characteristic which is stable and consistent across referents, contexts and time. For instance, Mayer et al. (1995) describe TP as a “stable within party factor” (p. 715) and their interpersonal trust model positions TP as an independent variable, which is not influenced by contextual factors or the outcomes of trust decisions. In line with this, literature consistently identifies TP as a “stable individual difference” (Gill et al., 2005, p. 289; Van Dyne et al., 2000, p. 6), “stable variable” (Alarcon et al., 2016, p. 313); “stable disposition” (Bernerth and Walker, 2009, p. 218), “stable individual variable” that is distinct from “trust as a situational state” (Tremblay et al., 2013, p. 237). Similarly, trust propensity has been described as an “enduring trait related to temperament and genetics, which is not person, context or lifetime dependent” (Mooradian et al., 2006, p. 3; Ashleigh et al., 2012, p. 362). Empirical literature has taken its lead from here and examined TP only as an independent variable. Indeed, Peralta and Saldanha (2014) argue that as TP is unrelated to context or the behavior of others, it is largely unmanageable from an organizational perspective. In contrast, in the wider social sciences literature, TP is studied as a dependent variable predicted by economic (e.g., income quality, Bjørnskov, 2007), cultural (Uslaner, 2008), climatic (Kong, 2013), and biological factors (Zak et al., 2005; Sturgis et al., 2010). Interestingly, this literature also typically assumes that TP is highly stable and cemented during childhood.

However, the theory put forward by trust theorists for how TP is established suggests the possibility of a counterargument. Specifically, Rotter (1971) suggests TP is developed through a process of social learning and is informed by levels of trust in specific, particularized relationships. Analogous concepts are recognized to have both stable and unstable aspects, for instance, Sitkin and Pablo (1992) suggest that risk propensity is influenced by the outcomes of previous risk-taking. Experimental research suggests that TP is influenced by regulatory orientation, with prevention focused individuals reporting lower TP due to sensitivity to potential negative outcomes and information as well as increased vigilance (Keller et al., 2015). Regulatory focus itself consists of both relatively stable and momentary facets and the accessibility and salience of one’s orientation varies across situations (Higgins, 1997).

### TP as a Developmental Trait

TP can be positioned in the personality literature as a lower-level facet trait related to the general domain of agreeableness – a trait characterized by cooperativeness, courteousness and tolerance (Barrick and Mount, 1991). Lower-level facet traits are more specific and context dependent than the higher order, decontextualized traits of the five factor model of personality and have more proximal influences on behavior (Paunonen and Ashton, 2001). As such, TP is more specific than agreeableness and has a direct link to individuals’ trust and behavior in relationships, although is not specific to any one relationship.

By their very definition, personality traits are relatively stable, enduring patterns of thoughts, feelings and behaviors that change little over time (Allport, 1937; Costa and McCrae, 1994). More recent advances, however, have demonstrated developmental changes in traits highlighting that the treatment of traits as consistent over time is entirely flawed at worst or an over-simplification at best. In a meta-analysis of longitudinal studies, Roberts et al. (2006) reported significant mean-level changes in an array of traits across the lifespan and that, largely, these changes are positive, with people becoming more socially dominant, conscientious and emotionally stable as they age. This evidence dismisses the notion that personality stabilizes by 30 years of age, instead pointing to continuing plasticity of traits well into old age. A far more limited body of research has considered whether experiences during more condensed periods of time allow for personality change. For instance, a study by Judge et al. (2014) demonstrated deviations from baseline personality tendencies over a 10 days period.

Although proponents of personality change have put forward various reasons for why personality might change, the scope of this paper does not allow for an account of each of these perspectives (see Roberts et al., 2008, for a full discussion). Our study is primarily grounded in the social investment principle theory, which argues that as individuals have to negotiate transitions into new social roles, they respond to environmental and interpersonal factors that fuel personality trait development (Roberts et al., 2005; Lodi-Smith and Roberts, 2007). Given the relational nature of TP and the workplace experiences we are studying, the theory that gives most attention to social experiences seems most appropriate. Furthermore, social investment theory is explicitly interested in transitional periods and, as such, is more sensitive to explaining change over shorter time periods than other approaches.

### Stability and Change in TP

Historically, constructs that are highly stable are said to be trait-like, whereas constructs that are relatively unstable are said to be state-like (Cronbach, 1957) but, it has long been recognized that most dimensions of psychological functioning have both continuous (stable) and discontinuous (unstable) components (Baltes, 1987; Hertzog and Nesselroade, 1987). Mathematical models of such psychological constructs using longitudinal data were initially developed in the 1990s (e.g., Steyer, 1989; Kenny and Zautra, 1995; Steyer et al., 1999) under the general rubric of latent state-trait (LST) theory (see McArdle, 2009; Steyer et al., 2015). These models permit the identification of (a) time-invariant trait components, (b) time-varying, situationally specific occasion components and (c) state components which reflect the combined influences of (a) and (b). Latent growth models (LGMs) have also proliferated as a powerful approach to modeling individual change over time (Grimm and Ram, 2018). A general and integrative approach that combines these two efforts, and one that we take advantage of in the present study, is Tisak and Tisak’s (2000) LC-LST model (see also Geiser et al., 2013). Based on the theoretical foundation of TP and the personality change literature, we used this state-of-the-science modeling approach to investigate:

Hypothesis 1: TP consists of situationally specific components in addition to the stable trait component.

### TP During Socialization

Personality theorists have argued that social role changes in young adulthood are particularly influential as commitment to new social roles related to establishing a career and/or a family is reinforced by the norms and expectations of society (Lodi-Smith and Roberts, 2007). The process through which individuals enter or re-enter into an organization is known as (re)socialization (Van Maanen and Schein, 1979). Socialization is a broader term used to refer to individuals joining a new organization or work group, while the term resocialization refers more specifically to those re-entering the workplace after a leave of absence, engaging in an expatriate assignment or experiencing a large scale corporate change project, such as a merger (Ladge and Greenberg, 2015). In either case, this period represents a significant transition in an individual’s work life and is accompanied by a period of uncertainty as they adapt to function effectively in their new environment.

As individuals adapt to new social environments, certain behaviors are reinforced, others are punished and individuals’ own behaviors are magnified through monitoring themselves and observing others (Caspi and Roberts, 1999). The socialization experiences involved can have substantial effects on individual personalities, identities and values (e.g., Bardi et al., 2014), especially if they affect personal narratives that individuals develop to make sense of their experiences and themselves (McAdams and Olson, 2010) as part of identity formation and reformulation. Moreover, commitment to a new social role involves investment in relationships with other individuals in the new social network and the acceptance of the behavioral norms and expectations associated with that role (Wood and Roberts, 2006). In the case of TP, for instance, commitment to a new social role as an employee in a particular organization may be associated with expectations to interact and cooperate with other members of the same organization. Generalized tendencies to trust others are likely to be particularly salient in this type of situation as commitment to the new role necessitates interaction with specific unknown other parties where issues of trust may be prominent. Changes in TP at this time are likely to be driven by adoption of the new social role of “employee” which prescribes a certain level of trust that is consistent with organizational norms and the norms and behavior of the employee’s new network of coworkers.

The population of interest in Study 1 is accounting graduates being socialized into an organization. Transitioning into a new role is a time of uncertainty, anxiety and adjustment, where interactions with organizational insiders have an enormous impact on new employee behavior (Nifadkar and Bauer, 2016; Allen et al., 2017). Empirical research provides evidence for changes in personality during these transitions. For instance in a longitudinal study of job beginners, changers and stayers, Denissen et al. (2014) studied relationships between personality and job characteristics and provide evidence of socialization effects for stayers, whose personalities adapted to the demands of the job. Although research has not examined the impact of socialization on TP, cross-sectional studies indicate that other life transitions may be impactful. Specifically, TP is lower in individuals in particular occupations (Alesina and La Ferrara, 2002) and the unemployed (Brehm and Rahn, 1997). Furthermore, socialization research suggests that relationship building, social acceptance and cognitive framing of the behavior of others are central to adaptation to the workplace (Ashford and Black, 1996; Wanberg and Kammeyer-Mueller, 2000). A combination of the salience of cooperation with colleagues while adjusting to the uncertainties inherent in a new workplace environment is likely to lead to instability in TP during this time.

While the vast majority of literature assumes that personality change is something that occurs gradually over long periods of time, recent meta-analytic findings have challenged this (Roberts et al., 2017). Theorists have begun to consider a punctuated equilibrium model of personality trait change that models change as happening more quickly, followed by a period of stability (Roberts et al., 2017). The model was designed to explain change in response to clinical interventions, however, this type of intervention has parallels with career transitions. Socialization represents an intense period of change in a new employee’s career, where expectations for interaction with others form rapidly and become more stable over time (Liden et al., 1993; Chen and Klimoski, 2003). Therefore, we expect that change in TP will be greater immediately after the new joiners begin the socialization process and that the rate of change will level off once they are assimilated into the organization.

*Hypothesis 2*: Change in TP will be greater immediately following the transition and will level off thereafter.

## Study 1 – Materials and Methods

### Participants

Participants in Study 1 were 204 new employees in the Irish practice of a Big 4 international accountancy and consultancy firm. The average age of the sample was 22.61 years (SD 1.23) and participants were 54.9% female. All participants had completed their Bachelor’s degree with a further 34.3% completing Masters level courses.

### Measures

TP was measured using six items from a seven item scale by Jarvenpaa et al. (1998). Participants responded using a 7-point Likert scale. Items were adapted slightly to suit the workplace context of this study, in line with previous studies that have reported acceptable Cronbach’s alphas (0.71; van der Werff and Buckley, 2017; and 0.80; Yakovleva et al., 2010). A sample item from the adapted scale is “Most people can be counted on to do what they say they will do.” In our sample the Cronbach’s alphas were acceptable across all three time points (T1 = 0.71; T2 = 0.73; T3 = 0.72).

### Procedures

Respondents in Study 1 began their new job for the organization on the same day. A total of 204 participants was invited to take part in the research. 195 responses were received at Time 1, 189 at Time 2 and 167 at Time 3, a response rate of 96, 93, and 82% respectively. Data were collected via a paper and pencil survey on their first day, 3 months after they joined and 1 year after they joined. The timing of the data collection is an important aspect of the study design. The interval between the first two waves represents the typical theoretical timeframe for initial socialization (Chen, 2005), offering an opportunity to assess the extent to which TP changes during this transition. The timing of the third data point allows us to ascertain whether this change was temporary or lasting.

### Analysis

We created three manifest indicators for a TP latent variable using item parcels at each measurement wave to ensure the measurement model was locally and globally identified. Item parcels result in higher indicator reliability, more nearly continuous data distributions, more parsimonious measurement models, fewer dual factor loadings, and less sampling error than individual items (Little et al., 2002). Preliminary analyses indicated that Item #4 in Jarvenpaa et al.’s (1998) measure was essentially uncorrelated with the remaining items. Consequently, we discarded this item and randomly allocated the remaining six items to three two-item parcels (Hall et al., 1999; Bandalos and Finney, 2001). Descriptive statistics and correlations are shown in Table 1. Correlations were generally higher within measurement waves than across waves, lending initial support for convergent and discriminant validity. Also, Ms and SDs suggested the absence of ceiling or floor effects and range restriction.

**TABLE 1:** Study 1 descriptive statistics and variable correlations.

	**1**	**2**	**3**	**4**	**5**	**6**	**7**	**8**	**9**
1. TP11	(0.44)								
2. TP12	0.35^∗^	(0.53)							
3. TP13	0.30^∗^	0.56^∗^	(0.58)						
4. TP21	0.27^∗^	0.28^∗^	0.24^∗^	(0.21)					
5. TP22	–0.02	0.26^∗^	0.26^∗^	0.38^∗^	(0.53)				
6. TP23	0.06	0.26^∗^	0.19^∗^	0.32^∗^	0.61^∗^	(0.74)			
7. TP31	0.34^∗^	0.18^∗^	0.10	0.36^∗^	0.13	0.27^∗^	(0.07)		
8. TP32	0.13	0.33^∗^	0.24^∗^	0.32^∗^	0.35^∗^	0.27^∗^	0.35^∗^	(0.64)	
9. TP33	0.14	0.35^∗^	0.31^∗^	0.28^∗^	0.37^∗^	0.38^∗^	0.37^∗^	0.61^∗^	(0.66)
Mean	4.21	4.44	4.04	4.28	4.80	4.42	4.18	4.59	4.16
SD	0.97	0.98	1.00	0.89	0.92	1.06	0.86	0.99	1.07

**Coefficients alpha are on the main diagonal. N ranges between 154 and 195. ^∗^p < 0.05.**

Using LISREL 8 (Jöreskog and Sörbom, 1993), we first assessed TP parcels’ measurement invariance over the three measurement waves according to Vandenberg and Lance’s (2000) paradigm. Next, we assessed Trait vs. Occasion variance components of TP and analyzed the form of change using Tisak and Tisak’s (2000) LC-LST model. In each step we used LISREL’s expectation-maximization (EM) algorithm for the treatment of missing data to provide initial estimates for full information maximum likelihood (FIML) estimation.

## Study 1 – Results

### Measurement Invariance (MI)

We input the 9 × 9 matrix of covariances among the three TP parcels at three waves for tests of MI using Vandenberg and Lance’s (2000) augmented covariance matrix approach. An omnibus test of the equality of covariance matrices demonstrated excellent fit [χ^2^(22) = 30.38, *p* = 0.11, CFI = 0.981, RMSEA = 0.043, 90% CI (0.0; 0.078)]. Thus, in this case of failing to reject the omnibus null hypothesis of no measurement differences over time “further tests of specific aspects of ME/I are neither needed nor warranted” (Vandenberg and Lance, p. 36). As such, we proceeded directly to tests of stable trait vs. occasion-specific aspects of TP.

### LC-LST Model

**FIGURE 1:**
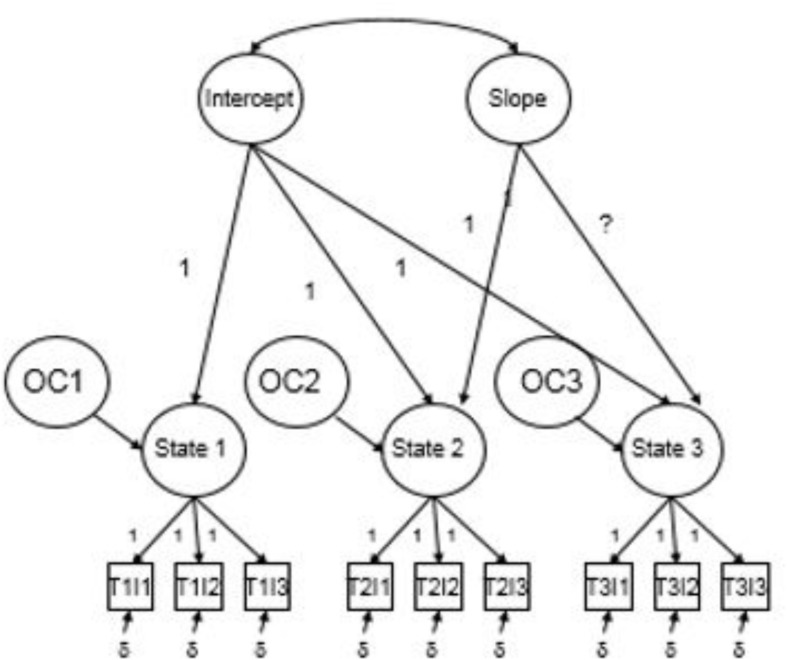
Generic LC-LST model.

A generic representation of Tisak and Tisak’s (2000) LC-LST model is shown in Figure 1. Conceptually, the model begins with a first-order factor (FOF) measurement model that fits a unidimensional structure to the three TP parcels at each measurement wave corresponding to the TP States. However, unlike conventional confirmatory factor analytic (CFA) measurement models in which factor loadings are freely estimated, factor loadings are fixed to unity in order to pass the observed covariance structure among the TP parcels up to the level of the first-order State factors for the purpose of partitioning into State, Trait and Occasion variance components and modeling longitudinal change in TP. A congeneric measurement structure is usually imposed, such that equality constraints are imposed on indicators’ uniquenesses both within and across measurement waves. Each State factor is a function of two SOFs representing the stable Trait component of TP (“initial status” or “intercept” in traditional LGM nomenclature) and Slope that models longitudinal change. The State residuals (shown as OC1 through OC3 in Figure 1) represent the situationally-specific Occasion factors associated with each measurement wave. The Intercept and Slope SOF loadings are fixed and State variance is fixed at 0 so that it is partitioned entirely into Intercept, Slope and residual/Occasion variance. As such, the model in Figure 1 partitions State variance associated with Year_j_ into a stable Intercept/Trait component, a coherent Slope component and less stable, situationally-specific Occasion components (see Alessandri et al., 2013; Vecchione et al., 2014, for notable applications of the LC-LST model).

**FIGURE 2:**
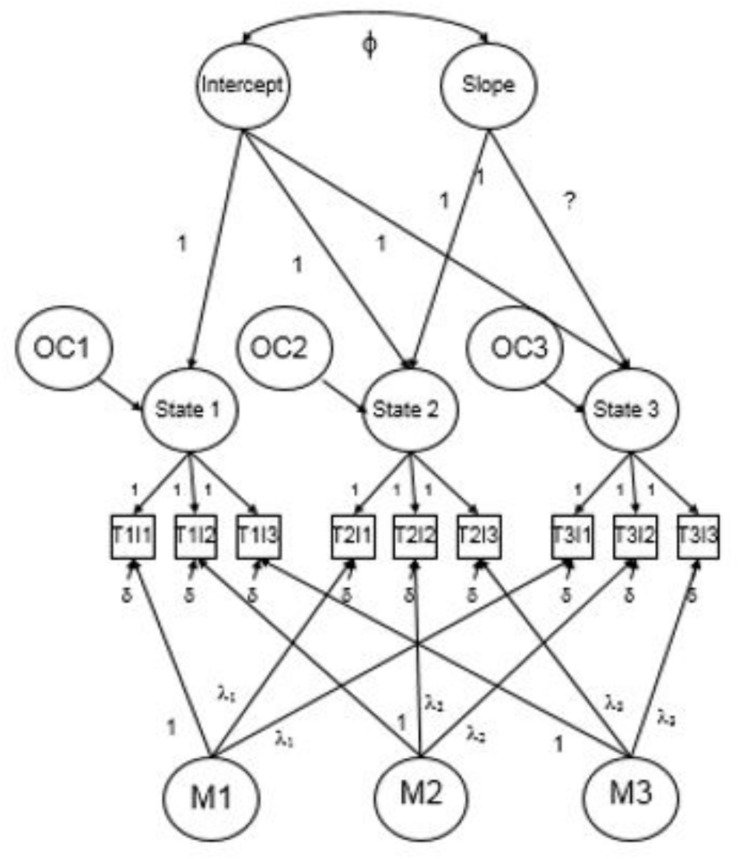
LC-LST model with orthogonal method effects.

LC-LST model selection results are summarized in Table 2. The basic LC-LST model shown in Figure 1 (Model 1) provided a poor fit to the data (Hu and Bentler, 1998, 1999). As suggested by LaGrange and Cole (2008), model fit can often be improved by adding Method factors to account for the repeated administration of the same measures. As such, Model 2 included three orthogonal Method factors corresponding to the three TP parcels (see Figure 2) and this improved model fit considerably [Δχ^2^(3) = 39.03 *p* < 0.01;ΔCFI ≥ 0.01 (Cheung and Rensvold, 2002); ΔRMSEA ≥ 0.015 (Chen, 2007)].^1^ Model 3 relaxed the assumption of congeneric measurement across indicators but this did not improve model fit. As a result, the more constrained congeneric measurement model was retained. Finally, Model 4 tested whether freeing the third basis coefficient to be estimated (i.e., an optimal Slope function) would improve model fit and it did not and so we retained the fixed coefficients for the linear model (i.e., 0, 1, 4). Thus, although the fit for all models failed to meet strict criteria for good fit, Model 2 was determined to be the most plausible model among those tested.

**TABLE 2:** Study 1 – LC-LST model selection.

**Model**	**df**	**χ^2^**	**RMSEA**	**CFI**	**NCSI**
1. Basic LC-LSTModel	40	124.76^∗∗^	0.10	0.75	0.80
1 vs. 2	3	39.03^∗∗^	0.02	0.07	
2^a^. Model 1 with Orthogonal Method Factors	37	85.73^∗∗^	0.08	0.82	0.88
2 vs. 3	2	4.19	<0.01	<0.01	
3. Model 2 with Heterogeneous Uniquenesses	35	81.54^∗∗^	0.08	0.82	0.89
2 vs. 4	1	0.07	<0.01	0.01	
4. Optimal Slope	36	85.66^∗∗^	0.08	0.81	0.88

**df, model degrees of freedom; RMSEA, Root Mean squared error of approximation (Cudeck and Browne, 1983); CFI, Comparative fit index (Bentler, 1990); NCSI, Non-centrality structural covariance index (O’Boyle and Williams, 2011). ^*a*^Best fitting model. ^∗∗^p < 0.01.**

Table 3 shows results of model variance decomposition at the level of the item parcels. State and Method variance components were calculated as the squared standardized SOF loadings; unique variance equals the estimated standardized uniqueness. On the average, 46% of the indicator variance was true score variance, 9% was method variance and 45% was unique variance. Thus, indicator validity = 0.46, reliability was = 0.55 and 9% Method variance, far below the amount of method variance that is commonly observed in the organizational and social sciences (Lance et al., 2010). Further decomposition of the State factor variance indicated that, on average, 41% was time-invariant Trait variance and 59% was time-varying Occasion variance. Thus, and in strong support of H1, TP was partially Trait-like but also had sizeable Occasion-specific variance components (see Table 4).

**TABLE 3:** Study 1 – Indicator-level percent variance decompositions.

**Indicator/Time**	**State true score variance**	**Method variance**	**Unique variance**
I1 T1	0.40	0.18	0.42
I2 T1	0.48	0.01	0.51
I3 T1	0.45	0.07	0.48
I1 T4	0.41	0.18	0.41
I2 T4	0.50	0.01	0.50
I3 T4	0.47	0.07	0.46
I1 T5	0.44	0.17	0.39
I2 T5	0.52	0.01	0.47
I3 T5	0.49	0.07	0.44
Mean	0.46	0.09	0.45

**TABLE 4:** Study 1 – TP state true-score variance decomposition.

**State**	**Trait variance**	**Occasion variance**
Time 1	0.44	0.56
Time 2	0.41	0.59
Time 3	0.37	0.63
Mean	0.41	0.59

Focusing on the change portion of the model, we found a non-significant estimate for the mean Slope factor (*M* = -0.02, *t* = 0.93) as well as its variance (*V* = 0.005, *t* = 0.74). Thus, although there was significant variability in TP Intercepts across participants (*V* = 0.18, *t* = 3.41) there was no significant mean change, nor significant differences in change patterns over time. Thus, H2 was not supported.

## Study 1 – Discussion

Study 1 set out to establish whether TP is less stable than previously thought and whether it displays situationally specific aspects. The results confirmed these predictions and demonstrated that although mean levels of TP remained stable, the construct had very sizable components of occasion or state related variance. Our results are noteworthy in that they demonstrate that TP is not wholly stable and trait like and so has the potential to change over time and to be influenced by antecedent variables. This is consistent with the social investment principle in that it shows that the exogenous shock of career transition can trigger a significant period of instability as an individual adapts to a new social role.

While these results contribute to the literature by challenging the treatment of TP as a stable, trait-like variable, there are some limitations to be noted. Firstly, Study 1’s population was a largely homogenous group of novice accountants, embarking on their first jobs within one firm. It could be argued that instability in traits is more pronounced at this life stage (Robins et al., 2001) and, thus state components of TP, as with other variables, are more likely. Furthermore, while we capture baseline levels of TP on their first day on the job, the design fails to capture pre-intervention levels of TP, that is, before socialization starts. In conducting our second study, we aim to address these issues and extend the scope of our research, by considering the potential correlates with other theoretically relevant variables.

To strengthen our findings, we designed a second study that compliments the first study in a number of valuable ways. First, it aims to replicate the finding that TP displays trait and situationally specific components. Second, it examines a more heterogeneous population by exploring TP changes in working mothers – while they are all women returning from maternity leave – they hold a diverse range of jobs in a variety of organizations. In doing so, any instability in TP cannot be solely attributed to the specific (re)socialization processes in one organization. The working mothers also represent a slightly later life stage, where some would argue that changes in traits are less pronounced (Costa and McCrae, 1994). Finally, we designed Study 2 to capture TP before the career transition to provide a baseline indicator of initial TP levels.

## Study 2 – Theory Development

Having established the existence of situational components of TP, we sought to investigate the levels of stability and change in TP in a second related population and over a longer period of time. Study 2 focuses on women resocializing into the workplace after maternity leave. While research on this specific population is rare, qualitative evidence indicates that this transition has a meaningful impact on self-concept and identity (Ladge and Greenberg, 2015). In line with Study 1, we argue that TP in this population will demonstrate both trait and situationally specific components. Furthermore, we expect that as these women resocialize into the workplace, change in TP will be centered on the immediate period around the transition after which it will be less malleable. Accordingly, we repeat our tests of Hypotheses 1 and 2 as outlined above.

### Potential Impact of TP Change

Our second study extends our focus by examining the impact of trait and situationally specific components of TP in a transitional population. The key theoretical argument for the influence of trust on work behavior is focused on cognitive resource allocation. Specifically, when high levels of trust are present, employees have the ability to focus cognitive resources on issues such as in-role and extra-role behavior (Mayer and Gavin, 2005). Meanwhile, low trust directs resources away from the job. Mayer and Gavin (2005) suggest that when employees lack trust, cognitive resources are likely to be spent on issues such as self-protection and defensive behavior. This is in line with conceptualizations of low trust that describe this state as being characterized by hesitance, a lack of confidence and, when combined with high distrust, vigilance, watchfulness and an attitude that the “best offense is a good defense” (Lewicki et al., 1998, p. 445).

The focus in the literature thus far has been on cognitive resources in relation to particularized trust in a specific other party. We extend this argument to suggest that generalized trust or TP will be an important predictor of cognitive resource use and depletion. As we have argued above, TP is particularly important in novel, ambiguous situations. Resocializing into the workplace after maternity leave is a transition characterized by uncertainty and brings a multitude of new social relationships to (re)establish. Individuals who have low TP are likely to expend more effort during this period in monitoring the behavior of others and protecting themselves against potential untrustworthy acts. When cognitive and self-control resources are expended on tasks such as these, self-regulation theories (e.g., Baumeister et al., 1998) suggest that individuals will experience a state of cognitive depletion and impaired capacity. Generalized, low expectations of others are likely to be even more impactful in this way than particularized trust which has been the focus of previous theoretical arguments. While low levels of trust in a particular relationship deplete resources when interacting with that specific coworker, the depletion is contained to this one relationship. In contrast, low levels of TP are likely to deplete resources in every workplace interaction. In contrast, individuals with high TP can be expected to experience no such depletion of resources.

The strength model of self-control and cognitive depletion (Baumeister et al., 1998) draws on a metaphor of muscle strength and fatigue to explain self-regulatory capacity. This metaphor is used to explain the phenomenon we mention above where by cognitively taxing tasks fatigue the muscle (Muraven and Baumeister, 2000; Baumeister et al., 2006). The metaphor has also been expanded to explain why there may be individual differences in the strength of the self-regulation muscle as a result of training by regularly engaging in takes that require self-control (Muraven et al., 1999; Gailliot et al., 2007). As such, we expect that individuals may have between person differences in propensity for cognitive depletion in addition to within person differences in state or situationally specific cognition depletion. In influencing the need for self-regulation in social interaction in specific situations and in a general outlook toward others, stable and situationally TP is likely to interact with these components of cognitive depletion. In line with this, we expect that:

Hypothesis 3: Stable and situationally specific components of TP are negatively related to stable and situationally specific components of cognitive depletion.

## Study 2 – Materials and Methods

### Participants

Participants were 247 women who were transitioning off maternity leave back to work. The average age of the sample was 33.81 years (SD 4.68). The largest single group at 43% was educated to Bachelor’s Degree level; 27% were educated to post-graduate or Masters level; 11% had their high school (Leaving Certificate) qualification. A further 5% had a Ph.D., 2.4% had a professional qualification and the remaining 9% had some post-secondary qualification. The participants were mostly working in professional/managerial roles (74.5%), with 22.2% working in lower-skilled jobs. Ninety two percent had been on leave for between 6 and 12 months, in line with policies that typically allow for 26 weeks of paid leave and unpaid leave for up to a year.

### Measures

Individual differences in TP were measured using a ten item scale from MacDonald et al. (1972). Sample items include “I expect other people to be honest and open.” The scale has demonstrated acceptable reliability in previous research (Baer et al., 2018b). The choice of a different measure of TP for Study 2 was driven by potential face validity issues for items that mention specific aspects of work such as projects or study for respondents who are drawn from a wider range of professions. Furthermore, by replicating results from Study 1 with a different measure of TP, we aim to provide additional support for the robustness of our findings. In this study, item 1 was essentially uncorrelated with the remaining items and was not included in the analysis. The Cronbach’s Alphas of the remaining nine items was acceptable across all three time points (T1 = 0.84; T2 = 0.86; T3 = 0.87). Cognitive depletion was measured using ten items from a twelve item scale by Ciarocco et al. (unpublished). Sample items include “I can’t absorb any more information.” Again, Cronbach’s alphas were acceptable across all three time points (T1 = 0.82; T2 = 0.88; T3 = 0.75).

### Procedures

Participants were invited through a number of channels; online social platform for mothers; our own networks and through large organizations that would have employees on maternity leave. We invited women to complete the first survey toward the end of their maternity leave, about 1 month before their planned return to work date. We invited the women to take an online survey. One month after their return to work, we sent a follow-up survey and again at 2 months after returning to work, we sent the final follow-up survey. Participation in the study was voluntary and the objectives were communicated at the outset of the study. There were issues with attrition over time and from the initial sample, response rates dropped to 45% at Time 2 and 28% at Time 3.

### Analysis

As in Study 1, we created three manifest indicators for a TP latent variable using item parcels at each measurement wave. Specifically, we randomly allocated MacDonald et al. (1972) scale items to three parcels with the constraint that each parcel contained three items (Hall et al., 1999; Bandalos and Finney, 2001). Analyses proceeded along the same lines as in Study 1 and once again we used LISREL’s EM algorithm for missing data to generate starting values for FIML estimation (Newman, 2014). Descriptive statistics and correlations among parcels are shown in Table 5. Correlations among parcels were again generally higher within measurement waves than they were across waves, providing support for their convergent and discriminant validity and Ms and SDs again suggested the absence of ceiling or floor effects and range restriction.

**TABLE 5:** Study 2 descriptive statistics and variable correlations.

	**1**	**2**	**3**	**4**	**5**	**6**	**7**	**8**	**9**
1. TP11	(0.71)								
2. TP12	0.69^∗^	(0.54)							
3. TP13	0.58^∗^	0.63^∗^	(0.69)						
4. TP21	0.43^∗^	0.30^∗^	0.52^∗^	(0.53)					
5. TP22	0.48^∗^	0.48^∗^	0.54^∗^	0.71^∗^	(0.61)				
6. TP23	0.53^∗^	0.46^∗^	0.64^∗^	0.73^∗^	0.64^∗^	(0.80)			
7. TP31	0.42^∗^	0.18	0.54^∗^	0.73^∗^	0.64^∗^	0.68^∗^	(0.73)		
8. TP32	0.41^∗^	0.30^∗^	0.49^∗^	0.67^∗^	0.70^∗^	0.74^∗^	0.79^∗^	(0.69)	
9. TP33	0.38^∗^	0.31^∗^	0.54^∗^	0.73^∗^	0.66^∗^	0.73^∗^	0.77^∗^	0.76	(0.68)
Mean	3.52	3.27	3.43	3.73	3.38	3.45	3.82	3.43	3.56
SD	0.82	0.76	0.94	0.68	0.77	0.99	0.69	0.74	0.79

**Coefficients alpha are on the main diagonal. N ranges between 45 and 175. ^∗^p < 0.05.**

## Study 2 – Results

### MI

Using the 9 × 9 matrix of covariances among the TP indicators, the omnibus test of equality of covariance matrices indicated that there was some (at least minor) lack of invariance over time [χ^2^(21) = 47.05, CFI = 0.93, RMSEA = 0.083, 90% CI 0.51; 0.11]. Consequently, we followed Vandenberg and Lance’s (2000) procedures for testing configural, metric and uniqueness invariance, and Yoon and Millsap’s (2007) approach using modification indices and Jung and Yoon’s (2016) approach based on parameter estimates’ confidence intervals to identify specific violations of MI. We found that parcel 1’s uniqueness at Time 1 was significantly larger, and that parcel 3’s uniqueness at Time 3 was significantly smaller than the same indicators at other measurement waves. Removing invariance constraints on these parameters produced a well-fitting model [Prenoveau, 2016; χ^2^(32) = 52.50, *p* > 0.01; CFI = 0.961; RMSEA = 0.056, 90% CI 0.28; 0.088]. As a result, we allowed these minor sources of lack of MI (i.e., heteroscedasticity) in formulating the LC-LST model.

### LC-LST Model

LC_LST model selection results are shown in Table 6. The addition of Method effects to the basic LC_LST model (Model 1 vs. Model 2) and allowing the heteroscedastic uniquenesses within measurement wave (Model 2 vs. Model 3) both improved model fit. Freeing the third Slope basis coefficient to be estimated did not improve model fit (Model 3 vs. Model 4) so that the selected LC-LST model was one that included orthogonal method effects associated with indicators over time, heterogeneous within-wave uniquenesses and a linear Slope coefficient (i.e., Model 3).

**TABLE 6:** Study 2 – LC-LST model selection.

**Model**	**df**	**χ^2^**	**RMSEA**	**CFI**	**NCSI**
1. Basic LC-LST Model (Figure 1)	38	109.01^∗∗^	0.10	0.81	0.86
1 vs. 2	3	37.99^∗^	0.02	0.05	
2. Model 1 with Orthogonal Method Factors	35	71.22^∗∗^	0.08	0.86	0.93
2 vs. 3.	2	20.08^∗∗^	0.02	0.04	
3^a^. Model 2 with Heterogeneous Uniquenesses	33	51.14^∗∗^	0.06	0.90	0.97
3 vs. 4	1	3.27	0.01	<0.01	
4. Optimal Slope	32	47.87^∗∗^	0.05	0.90	0.97

**df, model degrees of freedom; CFI, Comparative Fit Index; RMSEA, Root Mean Squared Error of Approximation*. *^*a*^Best fitting model. ^∗^p < 0.05, ^∗∗^p < 0.01.**

Table 7 shows TSO model variance decomposition at the level of the item parcels. On the average, 64% of the indicator variance was true score variance, 10% was method variance and 26% was unique variance. Thus, indicator validity = 0.64, reliability was = 0.74 and 10% Method variance. Further decomposition of the State factor variance (Table 8) indicated that, on average, 90% was time-invariant Trait variance and 10% was Time varying Occasion variance, providing additional support for H1 that TP would exhibit Occasion variance above and beyond stable Trait-like variance. Table 9 shows descriptive statistics for the change portion of the LC-LST model. TP was relatively high at time 1 (*M* = 3.56) and increased linearly across time, providing partial support for H2.

**TABLE 7:** Study 2 – indicator-level LC-LST model percent variance decompositions.

**Indicator/Time**	**State true score variance**	**Method variance**	**Unique variance**
I1 T1	0.69	0.03	0.28
I2 T1	0.71	0.01	0.28
I3 T1	0.54	0.24	0.22
I1 T4	0.67	0.03	0.30
I2 T4	0.69	0.01	0.30
I3 T4	0.51	0.26	0.13
I1 T5	0.70	0.03	0.27
I2 T5	0.72	0.01	0.27
I3 T5	0.55	0.24	0.13
Mean	0.64	0.10	0.26

**TABLE 8:** Study 2 – TP state true-score variance decomposition.

**State**	**Trait variance**	**Occasion variance**
1	0.90	0.10
2	0.89	0.11
3	0.91	0.09
Mean	0.90	0.10

**TABLE 9:** Study 2 – TP latent change descriptive statistics.

**Variable**	**Mean**	**VARCOVs**	**Λ’:**	**Y_1_**	**Y_2_**	**Y_3_**		
h_1_ – initial status	3.526^∗∗^	0.366^∗∗^		1.0^F^	1.0^F^	1.0^F^		
h_2_ – change	0.155^∗^	–0.081	0.136^∗∗^			0.0^F^	1.0^F^	1.5^F^

**VARCOVS, Variances and covariance. ^∗^p < 0.05, ^∗∗^p < 0.01, ^*F*^*, *Fixed parameter.**

### Bivariate LST Model

Preliminary scale analyses indicated that two of the cognitive deletion scale items (Item 5 “I feel calm and rational” and Item 8 “I feel sharp and focused” were essentially uncorrelated with the remaining scale items and so we deleted them from analysis. We re-assigned the remaining 10 items to three parcels containing 4, 3 and 3 items each that we used as manifest indicators for the Depletion latent variable. In order to test H3, we augmented a univariate TP LST model by fitting a bivariate LST model, where we simultaneously estimated Trait and Occasion variance components for both TP and Depletion together in a single combined model. The fit of this model was good [χ^2^(141) = 242.36, RMSEA = 0.06, 90% CI 0.05; –0.08]. Like TP, cognitive depletion also consisted of stable Trait variance (54%) and Occasion-specific variance (46%). More importantly, TP and cognitive depletion were inversely related in terms of their Trait (*r* = –0.33, *p* < 0.01) and Occasion (*r* = –0.55, *p* < 0.01) variance components, supporting Hypothesis 3.

## General Discussion

Given its implications in novel and ambiguous contexts, TP is gathering importance in the dynamic workplace of today (Frazier et al., 2013), where individuals are confronted with high levels of change in workplace structures and high levels of virtual work. This study seeks to establish the stability, or otherwise, of TP during career transitions, periods of social role investment such as starting a new job or returning to work after leave. These episodes represent ideal opportunities to apply the LC-LST model and demonstrate the potential of this model in advancing our knowledge of (in)stability in organizational constructs.

Across two field studies, our results demonstrate that TP is less stable than has been traditionally assumed. This has important implications and suggests that considerable future research is warranted to fully understand the ongoing influence of TP in the workplace. Our research suggests that TP is not necessarily an exogenous variable and may be influenced by important career transitions, particularly those that involve a change in the immediate social network. This possibility is not typically recognized by repeated measures in empirical research or in theoretical models that generally position TP as an antecedent (e.g., Mayer et al., 1995; McKnight et al., 1998). Our results suggest that when studying trust over time, researchers should strive to capture repeated measures of TP as baseline levels can no longer be assumed to remain stable. In particular, the pattern of results obtained suggest that instability and change in TP can be expected in the period surrounding a career transition. Further research is needed to establish the extent to which these changes are likely to restablize at a new level following the adjustment period.

Beyond demonstrating the existence of situationally specific components of TP, our research demonstrates changes in this generalized trust variable are important in that they are correlated with other variables, in this case, cognitive depletion. The dominant explanation for the impact of trust on performance and other workplace behaviors has been that high levels of trust leave cognitive resources free to be dedicated to tasks other than monitoring coworkers (Mayer and Gavin, 2005). This study provides evidence for a relationship between more generalized trust and cognitive depletion, such that stable and situationally specific aspects of both variables are significantly and negatively related. Our findings indicate significant relationships between the stable aspects of TP and cognitive depletion and between the situationally specific aspects of the same variables. We should acknowledge the possibility that the relationship between TP and cognitive depletion may be reciprocal. Although the dominant argument in the trust literature is that low levels of trust deplete cognitive resources (e.g., Mayer and Gavin, 2005), experimental studies have suggested that cognitive depletion leads to lower levels of cooperation in trust games (Ainsworth et al., 2014).

Our study also makes a contribution to the personality change literature. Almost the entire body of extant work on personality change has focused on lifespan developmental changes (e.g., Robins et al., 2001; Roberts et al., 2006), likely due to the widely held assumption that any changes in traits are slow and gradual. Notable recent exceptions on short-term change demonstrated the potential for personality changes to occur as a result of clinical interventions (Roberts et al., 2017) as well as in response to workplace experiences (Judge et al., 2014; Baer et al., 2018a). Our findings align with Roberts et al. (2017) and contribute to the personality change literature by extending on their meta-analytic findings in including two non-clinical populations who are negotiating naturally occurring transitions, rather than therapeutic interventions. The dynamic nature of TP we demonstrated extends beyond the daily variations reported by Judge et al. (2014) and Baer et al. (2018a) which return to baseline tendencies. Our results confirm that aspects of personality, in this case, TP, can change significantly in a relatively short period of time and that instability in TP can endure once the immediate impetus for the change has passed. Rather than demonstrating temporary shifts of a similar nature to Judge et al. (2014) and Baer et al. (2018a), our findings show that significant social transitions can have a lasting and meaningful impact on personality.

Our findings corroborate the idea that changes occur in early adulthood (Robins et al., 2001), both our samples are almost entirely drawn from populations between the ages of 20 and 40, though rather than showing that changes slowly evolve across this period, our results show notable changes occurring within a short period. Moreover, the results are in line with research on the social investment principle, where change in both samples occurred during transitions that involve social investments, which acted as catalysts for personality instability and/or change (Lodi-Smith and Roberts, 2007). Not only does the social investment trigger a transitory change as these individuals adjust, the findings support the idea that it results in a fundamental and lasting shift in TP that extends beyond the initial point of social investment and that this change impacts other theoretically relevant variables.

### Practical Implications

The results show that career transitions act as catalysts for changes in TP, thus serving as important contexts for influencing otherwise stable aspects of self. Those designing socialization or re-entry programs must be cognizant of the potential scope of such experiences in shaping individuals’ sense of self and, moreover, the aspects of self that determine how they relate to others. While the results here largely show increases or stable overall levels of TP, they do not negate the possibility of decreases in TP due to negative experiences. Thus, at the outset of a transition, managers and those responsible for supportive (re)socialization programs should clearly establish the expectations held by the individuals involved. Any damage done to TP during this time could have lasting effects on the quality of relationships with others in the organization going forward. Establishing a culture of open communication and providing mechanisms through which individuals involved in transitions can engage in positive relational interactions may enhance the potential for TP to increase during this critical phase.

### Limitations and Future Research

Proponents of the state-artifact issue (Nye et al., 2016) may argue that our measure of TP is contaminated by other state aspects of trust such that the TSO model is picking up on different aspects of the measure rather than showing the variable of TP as having state components. In demonstrating patterns of stability and instability across both studies, using different measures of TP, we aim to partially address these issues. Furthermore, the data are entirely self-report, though as a self-perceptual construct it would be difficult to capture TP through alternative methods (Chan, 2009).

Another methodological issue plaguing longitudinal research is reflected in the Study 2 attrition rate. More controlled, field experimental approaches to measuring changes in TP as a result of intervention might help to overcome these issues and would provide a greater capacity for isolating antecedents to TP changes. Furthermore, the timing of our data collection points may have impacted our results. As we failed to capture a pre-transition baseline in Study 1, we designed Study 2 to address this. However, future research might aim to capture TP levels before individuals engage in a recruitment process or take leave from the organization.

Future research is needed to explore the role of (in)stability in TP across a variety of contexts. We suggest that using baseline TP to predict variables at a later stage in a longitudinal study, may be diluting assessments of its longer term impact. Further research examining the impact of change in TP and its correlates will be vital in illuminating these issues and informing future theory development. Clearly, our findings are somewhat limited in relying on three data points only, the maximum follow up point being 1 year post-baseline in Study 1. It would be useful to establish the stability of these changes at further follow up points, well beyond the transitional experience. To this end, future research should examine the patterns of change we have observed using a variety of methodological approaches and that extend beyond the transitional contexts we examined in this study. In particular, we would encourage scholars to consider studying TP at more regular intervals during a transitional period to further illuminate patterns of change (see for instance the work of Omar Solinger and colleagues in the commitment and psychological contract field; e.g., Solinger et al., 2015, 2016). We also encourage scholars interested in the theoretical and empirical study of relationships during workplace transitions (e.g., socialization, new team formation, organizational mergers) to consider to the potential for reciprocal relationships between personality traits and experiences of change and ongoing employee behavior.

## Conclusion

Our research suggests that assumptions in the trust literature regarding the stability of TP are unwarranted and that trust studies should allow for the possibility that TP has situationally specific aspects and can change over time. We argue that TP may change during any period where a significant shift in an individual’s social network is experienced and the fact that the change in our studies was sustained over time suggests that such changes cannot be dismissed as transitory fluctuations. Career transitions such as (re)socialization to organizations, teams and other work groups are a pervasive aspect of modern organizational life. Our longitudinal studies of these transitional periods provide a first look at short-term personality change during a significant workplace event.

## Data Availability Statement

The datasets generated for this study are available on request to the corresponding author.

## Ethics Statement

The studies involving human participants were reviewed and approved by the Research Ethics Committee, Dublin City University. The patients/participants provided their written informed consent to participate in this study.

## Author Contributions

LW and YF designed the study and collected the data. FB was involved in the study design. CL conducted the data analysis. All authors contributed to the preparation of the manuscript.

## Conflict of Interest

The authors declare that the research was conducted in the absence of any commercial or financial relationships that could be construed as a potential conflict of interest.

## Footnotes

1 These factors have also been labeled as “variable specific factors” (Vecchione et al., 2014, p. 124). As the Method factors capture each parcel’s unique variances over time, variances associated with the ds in Figure 2 should be regarded as non-systematic error variances.
